# Frequency, proportion of PF-ILD, and prognostic factors in patients with acute exacerbation of ILD related to systemic autoimmune diseases

**DOI:** 10.1186/s12890-022-02197-3

**Published:** 2022-10-26

**Authors:** Noriyuki Enomoto, Hyogo Naoi, Yasutaka Mochizuka, Takuya Isayama, Yuko Tanaka, Atsuki Fukada, Yuya Aono, Mineo Katsumata, Hideki Yasui, Kazutaka Mori, Masato Karayama, Hironao Hozumi, Yuzo Suzuki, Kazuki Furuhashi, Tomoyuki Fujisawa, Naoki Inui, Yutaro Nakamura, Takafumi Suda

**Affiliations:** 1 Second Division, Department of Internal Medicine, Hamamatsu University School of Medicine, Hamamatsu, Japan; 2 Health Administration Centre, Hamamatsu University School of Medicine, 1-20-1 Handayama, Hamamatsu, 431-3192 Japan; 3 Medical &, Biological Laboratories Co., Ltd., Nagoya, Japan; 4 Respiratory Medicine, Shizuoka City Shimizu Hospital, Shizuoka, Japan; 5 Department of Clinical Pharmacology and Therapeutics, Hamamatsu University School of Medicine, Hamamatsu, Japan

**Keywords:** Acute exacerbation, Interstitial lung disease, Connective tissue disease, Systemic vasculitis, Idiopathic pulmonary fibrosis

## Abstract

**Background:**

Acute exacerbation (AE) of systemic autoimmune disease-related interstitial lung diseases (SAID-ILD) is less common than AE of idiopathic pulmonary fibrosis (IPF) and the details of AE-SAID-ILD have not been elucidated, but the prognosis is similarly devastating. This study was undertaken to determine the incidences of AE-ILD in each SAID and to elucidate the proportion of progressive fibrosing (PF)-ILD in AE-SAID-ILD.

**Methods:**

We retrospectively analysed data for patients with SAID-ILD who were diagnosed and observed at our hospital between 1999 and 2020.

**Results:**

Two hundred and thirty-two patients with SAID-ILD were enrolled, with a mean observation period of 100.2 months. AE-SAID-ILD was found in 25 patients (10.78%), mainly in patients with RA (17 patients, 68%) and elderly male patients with a smoking history. The overall incidence of AE-SAID-ILD was 1.29%/person-year, and the incidence for each SAID was as follows: RA 2.193, microscopic polyarteritis (MPA) 3.203, systemic sclerosis (SSc) 2.277, primary Sjögren syndrome 0.426, and polymyositis/dermatomyositis 0.222. The incidence of AE of RA/MPA/SSc-ILD was significantly higher than that of other AE-SAID-ILD (*p* < 0.001). Five of 25 patients (20%) fulfilled the criteria for PF-ILD. The 90-day survival rate was 48.0%, and a higher neutrophil count at AE (HR 13.27, 95%CI 2.447–246, *p* = 0.001) and early commencement of long-duration direct haemoperfusion with a polymyxin B-immobilised fibre column (HR 0.105, 95%CI 0.005–0.858, *p* = 0.035) were significant prognostic factors.

**Conclusions:**

The incidence of AE-SAID-ILD was significantly higher in patients with RA, MPA, or SSc than in patients with other SAID. Furthermore, even in patients with AE-SAID-ILD, the proportion of PF-ILD just before AE was not high (20%).

**Supplementary Information:**

The online version contains supplementary material available at 10.1186/s12890-022-02197-3.

## Introduction

Acute exacerbation (AE) of interstitial lung diseases (ILD) is a devastating condition that frequently occurs in patients with idiopathic pulmonary fibrosis (IPF), especially in its late stage [1–5]. AE-IPF accounts for 30%–40% of deaths in patients with IPF [6, 7], and is the most frequent cause of death in patients with IPF [7] or idiopathic interstitial pneumonia (IIPs) [8]. AE also occurs in other ILDs, such as idiopathic nonspecific interstitial pneumonia [9], unclassifiable IIP [10], connective tissue disease (CTD)-associated ILD [9, 11–13], and microscopic polyarteritis (MPA)-associated ILD [12].

Regarding systemic autoimmune diseases (SAID), including CTD and MPA, the presence of ILD has the significant impact on the long-term clinical course and the mortality [14, 15]. AE-ILD sometimes occurs during the clinical course in patients with SAID-ILD and makes the prognosis further worse than in those without AE-ILD [12, 14, 16, 17]. On admission of patients with AE-SAID-ILD, the exclusion of infectious diseases [18, 19] and drug-induced pneumonitis [20] is important to swiftly initiate proper treatments for saving patients with AE-SAID-ILD. AE-SAID-ILD is most common in patients with rheumatoid arthritis (RA) [11, 12], and was associated with decreased long-term survival of patients with RA during their clinical course [16, 17]. In addition, although the incidence of AE-ILD is relatively low in patients with SAID compared with IPF [9, 11], AE-SAID-ILD was associated with a poor prognosis after the onset of AE, similar to that of AE-IPF [12, 13]. However, little is known about the detailed incidence of AE-ILD in each SAID, or the proportion of progressive fibrosing (PF)-ILD associated with worsening ILD, even after treatment with corticosteroids and immunosuppressants [21]. Furthermore, no effective treatments for AE-SAID-ILD have yet been established. In the current study, we therefore retrospectively evaluated the incidences and clinical features of AE-SAID-ILD, as well as the prognostic factors, including treatments for AE-SAID-ILD. To the best of our knowledge, this is the first study to reveal the detailed incidence of AE-ILD for each SAID, the proportion of PF-ILD just before AE, and the possible therapeutic effect of direct haemoperfusion with a polymyxin B-immobilised fibre column (PMX-DHP) in patients with AE-SAID-ILD.

## Methods

### Study design and subjects

We retrospectively studied 232 patients with SAID-ILD, including CTD and MPA-related ILD, who were diagnosed and observed at our hospital between 1999 and 2020. The patients included 84 with RA, 51 with polymyositis/dermatomyositis (PM/DM), 28 with primary Sjögren syndrome (pSS), 17 with MPA, 16 with systemic sclerosis (SSc), nine with systemic lupus erythematosus (SLE), one with mixed connective tissue disease (MCTD), and 26 with several overlapping SAIDs (others). Patients with AE-SAID-ILD who had an acute, clinically significant respiratory deterioration characterized by new widespread alveolar abnormalities were selected. All patients met the modified diagnostic criteria for AE-SAID-ILD described by Collard et al. in 2016 [22]. Briefly, these included: 1) previous or concurrent diagnosis of ILD, 2) acute worsening or development of dyspnoea typically < 1 month in duration; 3) computed tomography (CT) with new bilateral ground-glass opacity and/or consolidation superimposed on background findings of ILD, and 4) deterioration not fully explained by cardiac failure or fluid overload. The study protocol was approved by the Ethics Committee of Hamamatsu University School of Medicine (approval number 18–085). All procedures in this study were performed in accordance with the study protocol and the 1964 Helsinki declaration as amended. The need for patient approval and informed consent was waived due to the retrospective nature of the study.

### Data collection

Clinical, laboratory, physiological, and treatment data were obtained from medical records. The severity of ILD within 12 months before the AE event was assessed using the gender, age, and physiology (GAP) staging system [23] and the Japanese Respiratory Society (JRS) severity grades for ILD [24]. The former considers gender, age, and lung physiology variables, forced vital capacity (FVC) and diffusion lung capacity for carbon monoxide (DLCO) [23]. The latter consists of PaO_2_ at rest and minimum SpO_2_ during a 6-min walking test (6MWT) [24]. All blood samples were collected on the first or second day of admission before starting treatments for AE-ILD. Serum concentrations of S100A8 were measured by an enzyme-linked immunosorbent assay with the cooperation of Medical & Biological Laboratories (MBL, Nagoya, Japan; CircuLex™ S100A8/MRP8 ELISA Kit). The extent of lung opacity was measured in three high-resolution CT (HRCT) slices, as described previously [25]. The pattern of AE-ILD on HRCT was classified as 1) peripheral, 2) multifocal, or 3) diffuse, as reported by Akira et al. [26]. The HRCT findings were reviewed by two observers. The frequency of AE-SAID-ILD was calculated by dividing the number of patients with AE-SAID-ILD by the total number of patients with SAID-ILD and observation period (%/person-year).

### Definition of PF-ILD

The patients were required to meet at least one of the following criteria for progression of ILD within the 24 months, despite standard treatment with an agent other than antifibrotic agents such as nintedanib or pirfenidone [21]: 1) a relative decline in the FVC of ≥ 10% of the predicted value, 2) a relative decline in FVC of 5% to < 10% of the predicted value and worsening of respiratory symptoms or an increased extent of fibrosis on HRCT, or 3) worsening of respiratory symptoms and an increased extent of fibrosis.

### Statistical analysis

Statistical analysis was performed using JMP-13.1.0 (SAS Institute Inc., Cary, NC, USA) and EZR 1.41 (Saitama Medical Centre, Jichi Medical University, Saitama, Japan) [27]. The occurrence of AE-SAID-ILD was estimated considering death before AE as a competing event, and analysed using Gray's method. The relationships between SAIDs and AE-SAID-ILD occurrence were evaluated by Fine-Gray tests. Overall survival was estimated using Kaplan–Meier curves. The relationships between variables and mortality were evaluated by Cox proportional hazards regression analysis. All tests were two-sided and statistical significance was set at *p* < 0.05.

## Results

### Incidence of AE-ILD in patients with SAID

**Fig. 1:**
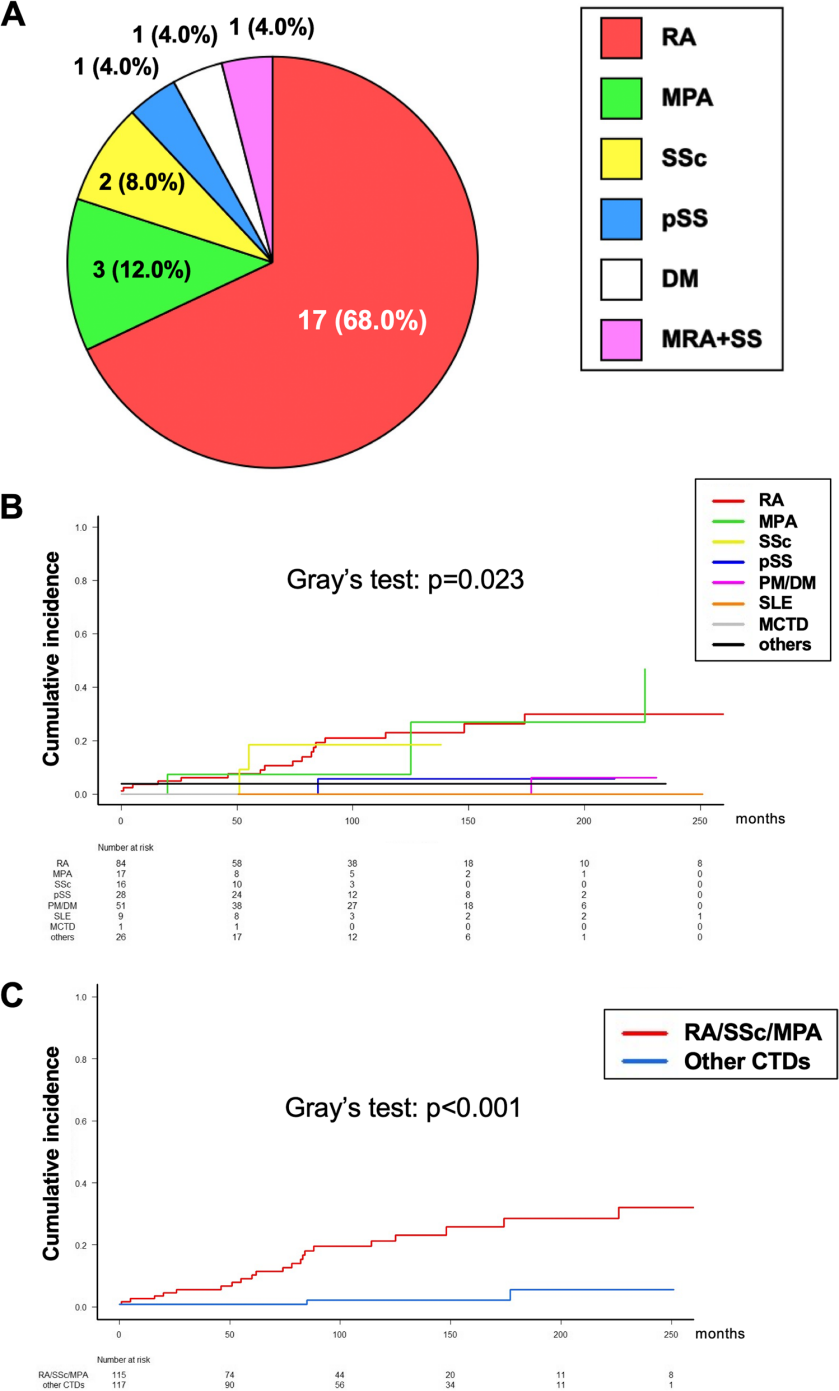
Breakdown of 25 patients with AE-SAID-ILD and incidence of each AE-SAID-ILD. A) Proportion of SAID among 25 patients with AE-SAID-ILD. B) Incidences of each SAID (Gray’s test, *p* = 0.023). C) Incidence of AE among patients with RA/MPA/SSc-ILD compared with other AE-CTD-ILD (Gray’s test, *p* < 0.001, Fine-Gray test; HR 8.324, 95%CI: 2.514–27.56). Abbreviations: AE-SAID-ILD: acute exacerbation of systemic autoimmune disease-related interstitial lung diseases, RA: rheumatoid arthritis, MPA: microscopic polyarteritis, SSc: systemic sclerosis, pSS: primary Sjögren syndrome, SS: secondary Sjögren syndrome, PM/DM: polymyositis/dermatomyositis, MRA: malignant RA, SLE: systemic lupus erythematosus, MCTD: mixed connective tissue disease, CTD: connective tissue disease, HR: hazard ratio

Twenty-five of the 232 patients (10.78%) with SAID-ILD had AE during the observation period (mean 100.2 months). The patient’s demographic, laboratory, physiologic, and radiologic data and treatments are shown in Table 1. The median age was 72 years, and males (17, 68%) and ex/current smokers (19, 76%) were predominant. Among the 25 patients with AE-SAID-ILD, the most common SAID was RA (17 patients, 68%) (Fig. 1), and other SAIDs included MPA (3 patients, 12%), SSc (2 patients, 8%), pSS (1 patient, 4%), DM (1 patient, 4%), and malignant RA + Sjögren syndrome (1 patient, 4%). The overall incidence of SAID-ILD was 1.29%/person-year, and the incidences of each SAID were as follows (Fig. 1B, Gray’s test, *p* = 0.023): RA, 2.193; MPA, 3.203; SSc, 2.277; pSS, 0.426; PM/DM, 0.222; SLE, 0; MCTD, 0; and several overlapping SAIDs (others), 0.481. The risks of AE were significantly higher in patients with RA-ILD and MPA-ILD compared with PM/DM-ILD (Fig. 1B, RA vs. PM/DM: Fine-Gray test, hazard ratio [HR] 11.35, 95% confidence interval (CI): 1.593–80.92; MPA vs. PM/DM: Fine-Gray test, HR 11.67, 95%CI: 1.365–99.81). The integrated incidence of AE of RA/MPA/SSc-ILD was significantly higher than that of other AE-CTD-ILD (Fig. 1C, Gray’s test, *p* < 0.001, Fine-Gray test; HR 8.324, 95%CI: 2.514–27.56).

**Table 1:** Demographic, laboratory, physiologic, and radiologic data and treatments in patients with AE-SAID-ILD

	**AE-SAID-ILD** (*n* = 25)
Age, year	72 (55, 85)
Sex, male / female	17 / 8
Smoking, never / ex / current	6 / 18 / 1
Pack-year of smoking	27.5 (0, 144)
Surgical lung biopsy, +/-	6 / 17
Period from ILD diagnosis to first AE, mo	74 (0, 226)
Period from SAID diagnosis to first AE, mo	64 (0, 365)
ILD preceding SAID, +/-	7 / 18
Observation period, day	60 (0, 228)
**Data before AE **^**a**^	
FVC, % pred	79.1 (49, 111)
DL_CO_, % pred	62.1 (39.3, 82.0)
PaO2 at rest, Torr	77.3 (61.3, 86.4)
UIP pattern on HRCT, +/- / unknown	12 / 10 / 3
Extent scores on HRCT (full score: 25)	12 (2, 20)
JRS severity grade, I / II / III / IV / unknown	6 / 0 / 3 / 1 / 15
The GAP staging system, I / II / III / unknown	8 / 3 / 0 / 14
Preceding immunosuppressive treatments, +/-	20 / 5
Preceding antifibrotic treatments, +/-	1 / 24
Preceding oxygen therapy, +/-	4 / 21
**Data at AE**	
Peripheral blood WBC, × 10^3^/μL	11.6 (7.4, 22.8)
Peripheral blood Neut, × 10^3^/μL	10.7 (6.3, 19.4)
Serum CRP, mg/dL	10.2 (1.5, 31.8)
Serum LDH, IU/L	405 (202, 815)
Serum KL-6, U/mL	1463 (340, 4341)
Serum SP-D, ng/mL	329 (74, 2950)
P/F ratio	180 (57, 328)
Extent scores on HRCT at AE (full score: 25)	18.5 (10, 25)
HRCT pattern at AE, peripheral / multifocal / diffuse / unknown	2 / 3 / 19 / 1
Administration of steroid pulse therapy ^**b**^, +/-	25 / 0
Duration from admission to starting treatments for AE, days	2 (0, 13)
Administration of Immunosuppressants, +/-	13 / 12
Treatment with PMX-DHP, +/-	8 / 17
Duration from admission to starting PMX-DHP, days	2 (0, 16)
Intubation, +/-	6 / 19

Data presented as median (range) or n (%)

*Abbreviations*: *AE* Acute exacerbation, *SAID-ILD* Systemic autoimmune disease-related interstitial lung disease, *FVC* Forced vital capacity, *DL*_*CO*_ Diffusion lung capacity for carbon monoxide, *UIP* Usual interstitial pneumonia, *HRCT* High-resolution computed tomography, *JRS* Japanese Respiratory Society, *GAP* Gender, age, and physiology, *PF-ILD* Progressive fibrotic interstitial lung disease, *WBC* White blood cells, *Neut* Neutrophils, *CRP* C-reactive protein, *LDH* Lactate dehydrogenase, *KL-6* Krebs von den Lungen-6, *SP-D* Surfactant protein D, *P/F* PaO_2_/FiO_2_, *PMX-DHP* Direct haemoperfusion with a polymyxin B-immobilised fibre column

^a^ Pulmonary function tests and severity scores were evaluated within 12 months before AE-SAID-ILD

^b^ Methylprednisolone 1,000 mg/day for 3 days

### Physiological examination findings, laboratory data, HRCT findings, and treatments

The clinical data for all patients with AE-SAID-ILD, including physiological examination, laboratory data, HRCT findings, and treatments within 12 months before the onset of AE-ILD, are shown in Table 1. Twelve patients (48%) had a usual interstitial pneumonia pattern on HRCT. High proportions of patients with AE-SAID-ILD were Grade 1 according to the GAP and JRS staging systems (32% and 24%, respectively). Twenty patients (80%) with AE-SAID-ILD received immunosuppressive treatments including corticosteroids and/or immunosuppressants, one patient (4%) received an antifibrotic agent, and four patients (16%) received oxygen therapy before AE. There were no patients who underwent surgical lung biopsy, other surgical operation, or mechanical ventilation immediately before AE-SAID-ILD. Regarding laboratory data at AE, the median PaO_2_/FiO_2_ (P/F) ratio was 180. The peripheral white blood cell (WBC) and neutrophil counts increased at AE (11.6 and 10.7 × 10^3^/μL, respectively). A diffuse pattern on HRCT at AE was the most frequent pattern (19, 76%) and the extent score on HRCT was 18.5/25. All of patients were treated with steroid-pulse therapy (methylprednisolone of 1,000 mg/day for 3 days) followed by a tapering dose of prednisolone. Thirteen patients (52%) received immunosuppressants, and eight (32%) received long-duration (mainly ≥ 12 h) PMX-DHP (Toray Medical Co., Ltd, Tokyo, Japan) [28, 29]. These treatments were commenced concomitantly with the antibiotics, as soon as possible after admission. There were no patients who underwent extracorporeal membrane oxygenation.

### Proportion of patients who fulfilled the criteria of PF-ILD

**Fig. 2:**
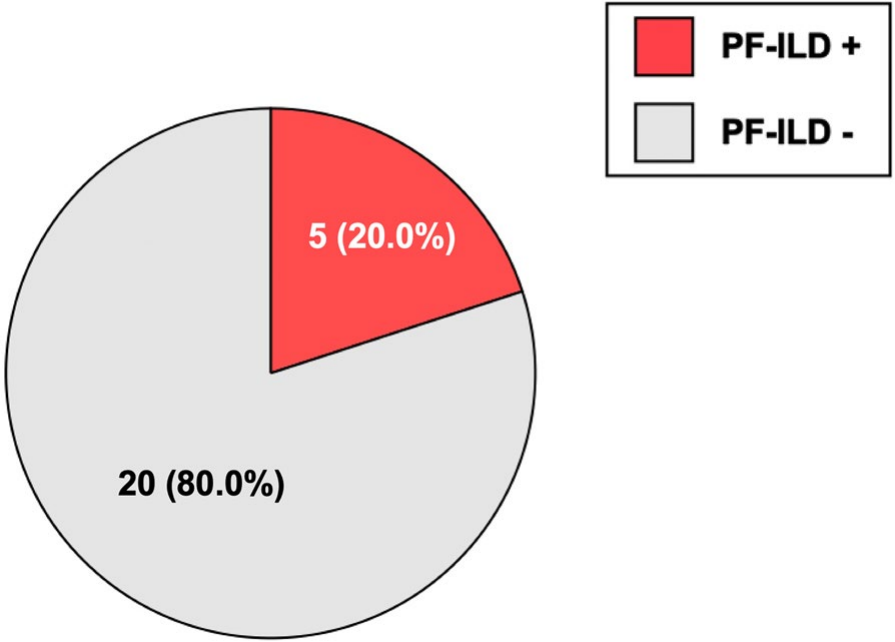
Proportion of patients with AE-SAID-ILD who fulfilled the criteria for PF-ILD within 12 months before AE. Abbreviations: PF-ILD: progressive fibrosing interstitial lung disease, AE-SAID-ILD: acute exacerbation of systemic autoimmune disease-related interstitial lung diseases

Of the 25 patients with AE-SAID-ILD, five (20%) fulfilled the criteria for PF-ILD [21] within 12 months before AE (Fig. 2). Among these patients, ILD progressed slowly before AE-ILD, despite standard treatments other than antifibrotic agents such as nintedanib or pirfenidone.

### Mortality rate and prognostic factors in all patients with AE-SAID-ILD

**Fig. 3:**
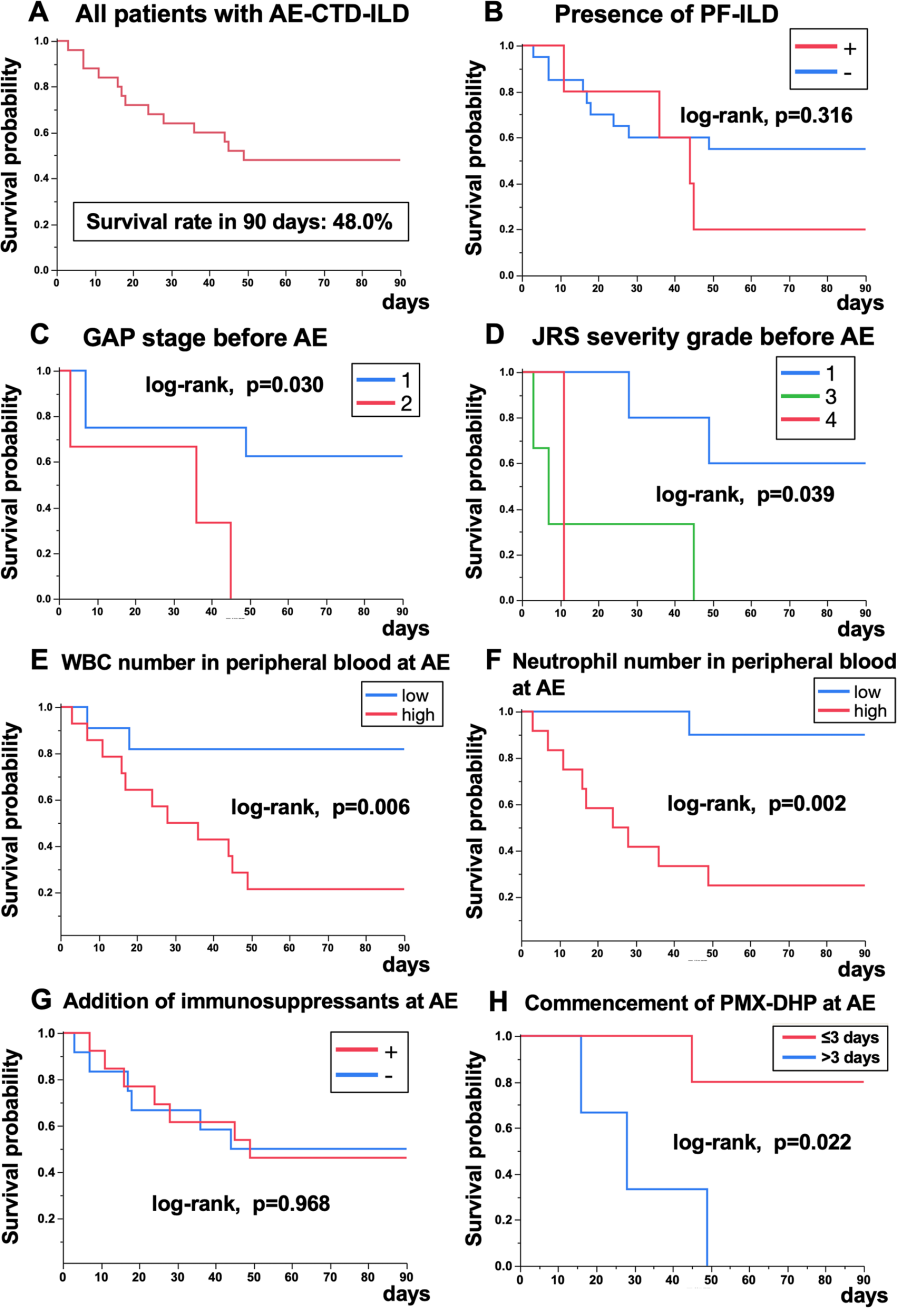
Kaplan–Meier survival curves in patients with AE-SAID-ILD. A) Ninety-day survival rate in patients with AE-SAID-ILD. B) Ninety-day survival was unaffected by the presence of PF-ILD (log-rank *p* = 0.316). Ninety-day survival according to C) GAP stage (*p* = 0.030) and D) JRS severity stage (*p* = 0.039) within 12 months before AE. Ninety-day survival in relation to E) peripheral WBC count (*p* = 0.006) and F) neutrophil count (*p* = 0.002). All patients received steroid-pulse therapy followed by a tapering dose of prednisolone. Ninety-day survival in relation to addition of G) immunosuppressants, mainly intravenous cyclophosphamide (*p* = 0.968) and H) PMX-DHP within 3 days after admission (*p* = 0.022). Abbreviations: AE-SAID-ILD: acute exacerbation of systemic autoimmune disease-related interstitial lung diseases, PF-ILD: progressive fibrosing interstitial lung disease, GAP: gender, age, and physiology, JRS: Japanese Respiratory Society, PMX-DHP: direct haemoperfusion with a polymyxin B-immobilised fibre column

Fifteen of the 25 patients died of respiratory failure, one died of lung cancer, one died of lower intestine bleeding, and one died of multiple cerebral embolisms during the whole observation period. The survival curves within 90 days after the onset of AE are shown in Fig. 3. Thirteen patients died within 90 days, and the 90-day survival rate after the onset of AE was 48% in patients with AE-SAID-ILD (Fig. 3A). Eleven of the 13 patients (84.6%) died of respiratory failure, one died of lower intestine bleeding, and one died of multiple cerebral embolisms within 90 days (Supplementary Fig. 1). Patient survival was not affected by the presence of PF-ILD (Fig. 3B, log-rank *p* = 0.316). Patients with the GAP stage 1 or JRS severity stage 1 before AE had significantly better survival (Fig. 3C, *p* = 0.030 and 3D, *p* = 0.039, respectively). Furthermore, a higher peripheral blood WBC count at AE (Fig. 3E, *p* = 0.006), and especially a higher neutrophil count (Fig. 3F, *p* = 0.002), were associated with significantly worse survival. However, survival was not related to serum S100A8, which is produced by activated neutrophils (*p* = 0.239, data not shown). Regarding treatments at AE, all of patients received steroid-pulse therapy followed by a tapering dose of prednisolone. The addition of immunosuppressants, mainly intravenous cyclophosphamide, did not improve survival (Fig. 3G, *p* = 0.968). In addition, treatment with PMX-DHP overall at AE did not significantly improve survival (*p* = 0.629, data not shown); however, commencement of PMX-DHP within 3 days after admission significantly improved the 90-day survival (Fig. 3H, *p* = 0.022).

We also evaluated prognostic factors using Cox proportional hazards models. The results of univariate Cox proportional hazards models of mortality in AE-SAID-ILD are shown in Table 2. JRS severity grade before AE (3/4 vs. 1/2, HR 10.97, *p* = 0.016) and peripheral blood WBC and neutrophil counts at AE (high vs. low based on median value, HR 6.350, *p* = 0.005 and HR 13.27, 95%CI 2.447–246, *p* = 0.001, *p* = 0.001, respectively) were significant prognostic factors. Serum S100A8 level at AE was not a significant factor (high vs. low, *p* = 0.239). Regarding treatments, the addition of immunosuppressants to corticosteroids was not a significant factor (*p* = 0.968), and although the treatment with PMX-DHP overall was not significant, commencement of treatment with long-duration PMX-DHP within 3 days after admission was a significant prognostic factor (HR 0.105, 95%CI 0.005–0.858, *p* = 0.035). Furthermore, serum lactate dehydrogenase 2 days after starting treatments for AE was a significant prognostic factor (HR 1.005, *p* = 0.006). Multivariate Cox proportional hazards models were not carried out due to a lack of statistical power.

**Table 2:** Cox proportional hazards models of mortality in patients with AE-SAID-ILD (*n* = 25)

Variable	Hazard ratio	95% CI		*p* value
		**lower**	**upper**	
Age, y	1.019	0.956	1.089	0.565
Sex, male	1.323	0.429	4.897	0.637
Smoking, never vs. former/current	0.851	0.191	2.789	0.805
Pack-years of smoking	1.011	0.992	1.030	0.250
SAID, RA vs. non-RA	2.127	0.648	9.514	0.225
Duration from SAID-diagnosis to AE, months	1.000	0.994	1.005	0.931
Duration from ILD-diagnosis to AE, months	1.005	0.996	1.014	0.279
ILD preceding SAID, +	1.521	0.458	4.580	0.472
**Data before AE **^**a**^				
FVC before AE, % pred	0.988	0.950	1.025	0.511
DL_CO_ before AE, % pred	0.980	0.908	1.046	0.545
HRCT pattern before AE, UIP vs. non-UIP	0.774	0.241	2.481	0.659
JRS severity grade before AE, 3/4 vs.1/2	10.97	1.535	219.6	0.016
GAP staging system before AE, 2/3 vs. 1	5.705	0.931	43.88	0.059
Preceding immunosuppressive treatments for SAID, +	4.066	0.798	74.14	0.101
Preceding oxygen therapy, +	2.071	0.461	6.857	0.305
PF-ILD, +	1.817	0.489	5.650	0.345
**Data at AE**				
P/F ratio at AE	0.994	0.986	1.003	0.208
Extent scores on HRCT at AE (full score 25)	1.120	0.972	1.322	0.120
HRCT pattern at AE, diffuse vs. non-diffuse	4.040	0.782	73.92	0.106
Peripheral blood WBC at AE, high (vs. low)	6.350	1.682	41.34	0.005
Peripheral blood Neutrophil at AE, high (vs. low)	13.27	2.447	246.2	0.001
Serum CRP at AE, mg/dL	1.002	0.999	1.005	0.218
Serum LDH at AE, U/L	1.002	0.999	1.005	0.240
Serum KL-6 at AE, U/mL	1.000	0.999	1.001	0.994
Serum S100A8 at AE, high (vs. low)	2.243	0.590	10.65	0.239
Treatments for AE within 3 days, +	0.435	0.141	1.609	0.194
Addition of immunosuppressants for AE, +	1.023	0.340	3.180	0.968
Treatment with PMX-DHP for AE, +	0.749	0.203	2.305	0.625
Treatment with PMX-DHP for AE within 3 days, +	0.105	0.005	0.858	0.035
Serum CRP 2 days after starting treatments for AE, mg/dL	1.086	0.999	1.169	0.051
Serum LDH 2 days after starting treatments for AE, U/L	1.005	1.001	1.008	0.006

*Abbreviations*: *AE* Acute exacerbation, *SAID-ILD* Systemic autoimmune disease-related interstitial lung disease, *RA* Rheumatoid arthritis, *FVC* Forced vital capacity, *DL*_*CO*_ Diffusion lung capacity for carbon monoxide, *UIP* Usual interstitial pneumonia, *HRCT* High-resolution computed tomography, *JRS* Japanese Respiratory Society, *GAP* Gender, age, and physiology, *PF-ILD* Progressive fibrotic interstitial lung disease, *WBC* White blood cells, *Neut* Neutrophils, *CRP* C-reactive protein, *LDH* Lactate dehydrogenase, *KL-6* Krebs von den Lungen-6, *SP-D* Surfactant protein D, *P/F* PaO_2_/FiO, *PMX-DHP* Direct haemoperfusion with a polymyxin B-immobilised fibre column

^a^ Pulmonary function tests and severity scores were evaluated within 12 months before AE-SAID-ILD

## Discussion

In the present study, we retrospectively evaluated 232 patients with SAID-ILD, including CTD- and MPA-related ILD, of whom 25 had AE-SAID-ILD. Among these 25 patients, RA was the most common baseline disease (68%), and the frequency of AE was significantly higher among patients with RA/MPA/SSc-ILD compared with other AE-SAID-ILD. The proportion of patients fulfilling the criteria for PF-ILD was 20%, and the 90-day survival rate was as low as 48.0%. Furthermore, more-severe ILD before AE, a higher neutrophil count at AE, and early commencement (within 3 days after admission) of long-duration PMX-DHP were significant prognostic factors. To the best of our knowledge, this study provides the first detailed evidence for the incidence of each AE-SAID-ILD and the proportion of PF-ILD in patients with AE-SAID-ILD.

Most of the 25 patients with AE-SAID-ILD were elderly males with an ex/current smoking history, in contrast to patients with common CTDs who tend to be young, females with a no-smoking history. Although the total reported incidence of AE-CTD-ILD or AE-SAID-ILD is lower than that of AE-IPF [9, 11–13], the incidences of each AE-SAID-ILD remain unclear because of their rarity. Furthermore, even among patients with IIPs, the incidence of AE-ILD was significantly lower among patients who met the criteria for interstitial pneumonia with autoimmune features (IPAF) compared with those without IPAF [8]. Autoimmune features may thus prevent exacerbation of ILD in both SAID and IPAF. In the present study, the incidence of AE of RA/MPA/SSc-ILD was significantly higher than that of other AE-SAID-ILD. Regarding the disease stage at which AE-ILD occurs, we previously reported that patients with AE-SAID-ILD had a significantly higher %FVC before AE compared with AE-IPF patients [12], suggesting that AE-SAID-ILD appears suddenly even in its earlier stage compared with patients with AE-IPF, although the mechanism remains unclear.

In terms of PF-ILD, the term progressive pulmonary fibrosis (PPF) was also recently proposed in international guideline [30] and PF-ILD/PPF are attracting attention. While most IPF patients show slow disease progression and IPF is the most representative disease of PF-ILD [31], PF-ILD accounts for 24%-31% in patients with CTD-ILD [32, 33]. However, the proportion of PF-ILD in the current study was not particularly high (20%), even in patients with AE-SAID-ILD. These results suggest that PF-ILD may not be strongly related to the occurrence of AE-SAID-ILD.

AE-SAID-ILD is deemed to be related to high levels of inflammation. Patients with AE-SAID-ILD accordingly had higher neutrophil counts and higher C-reactive protein at AE compared with patients with AE-IPF. This high inflammatory status mimic infectious diseases, leading to a delay in the appropriate treatments for AE-ILD [12]. The peripheral blood neutrophil count and CRP were apparently high at AE in the current study; however, the addition of immunosuppressants, mainly intravenous cyclophosphamide, to corticosteroid-pulse therapy did not improve the poor prognosis. The addition of cyclophosphamide to corticosteroids also failed to improve the prognosis in patients with AE-IPF [34, 35]. Novel treatments, other than immunosuppressants, are therefore warranted for the treatments of AE-ILD. In the pathogenesis of AE-IPF, neutrophils infiltrate the alveolar wall and play a role in the progression of diffuse alveolar damage, especially in the very early phase of AE-IPF [22, 36]. Treatment with long-duration PMX-DHP was previously shown to remove activated neutrophils [37] and improve survival in patients with AE-IPF [28, 29, 38] or AE-unclassifiable IIP [10]. In the current study, early commencement (within 3 days after admission) of long-duration PMX-DHP also significantly improved survival. Long-duration PMX-DHP thus seems to be a novel therapeutic option for AE-SAID-ILD. In addition, antifibrotic agents, such as nintedanib and pirfenidone, may also be novel treatments for AE-ILD, given that baseline antifibrotic therapy decreased the incidence of AE-IPF [39, 40], and improved survival after the onset of AE-IPF [34, 40]. These new treatments may improve the currently poor prognosis of patients with AE-ILD better than immunosuppressants.

This study had several limitations. First, the number of patients with AE-SAID-ILD was small because of the rarity of the condition. Second, the data were collected retrospectively. Finally, the treatments for AE-SAID-ILD varied, and notably, only one patient in the study received an antifibrotic agent before AE-SAID-ILD. A larger prospective study is therefore needed to assess the precise effects of treatments on AE-SAID-ILD.

In conclusion, we retrospectively evaluated 232 patients with SAID-ILD for a long period, and found 25 patients with AE-SAID-ILD. AE was significantly more common among patients with RA/MPA/SSc-ILD compared with other AE-SAID-ILD. The proportion of patients fulfilling the criteria for PF-ILD was 20%. More-severe ILD before AE, a higher neutrophil count at AE, and early commencement (within 3 days after admission) of long-duration PMX-DHP were significant prognostic factors. These findings imply that more attention should be paid to AE-ILD in patients with RA, MPA, or SSc, and the removal of activated neutrophils may be a promising treatment for improving respiratory condition in patients with AE-SAID-ILD. This information should aid the management of patients with AE-SAID-ILD. However, further prospective studies are needed to identify effective treatments, such as PMX-DHP and antifibrotic agents, to improve the currently poor survival of patients with AE-SAID-ILD.

## Supplementary Information

**Additional file1: Supplementary Fig. 1.** Causes of death within 90 days after the onset of AE-SAID-ILD. Thirteen patients died within 90 days. Eleven of the 13 patients (84.6%) died of respiratory failure, one died of lower intestine bleeding, and the other  died of multiple cerebral embolisms within 90 days. Abbreviations: AE-SAID-ILD: acute exacerbation of systemic autoimmune disease-related interstitial lung diseases.

## Data Availability

The data underlying this article cannot be shared publicly due to the protection of privacy in individuals that participated in the study. The data will be shared on reasonable request to the corresponding author.

## Abbreviations

AE: Acute exacerbation
CTD-ILD: Connective tissue disease-related interstitial lung disease
CRP: C-reactive protein
DL_CO_: Diffusion lung capacity for carbon monoxide
FVC: Forced vital capacity
GAP: Gender, age, and physiology
HRCT: High-resolution computed tomography
JRS: Japanese Respiratory Society
KL-6: Krebs von den Lungen-6
LDH: Lactate dehydrogenase
MPA: Microscopic polyarteritis
Neut: Neutrophils
P/F: PaO_2_/FiO_2_
PF-ILD: Progressive fibrotic interstitial lung disease
PM/DM: Polymyositis/dermatomyositis
PMX-DHP: Direct haemoperfusion with a polymyxin B-immobilised fibre column
pSS: Primary Sjögren syndrome 0.426
RA: Rheumatoid arthritis
SAID: Systemic autoimmune disease
SP-D: Surfactant protein D
SSc: Systemic sclerosis
UIP: Usual interstitial pneumonia
WBC: White blood cells
