# Supplementary figures and images for: Frequency, proportion of PF-ILD, and prognostic factors in patients with acute exacerbation of ILD related to systemic autoimmune diseases
Source: BMC Pulm Med. 2022 Oct 26;22:387. doi: 10.1186/s12890-022-02197-3 (PMC9608932; doi:10.1186/s12890-022-02197-3)

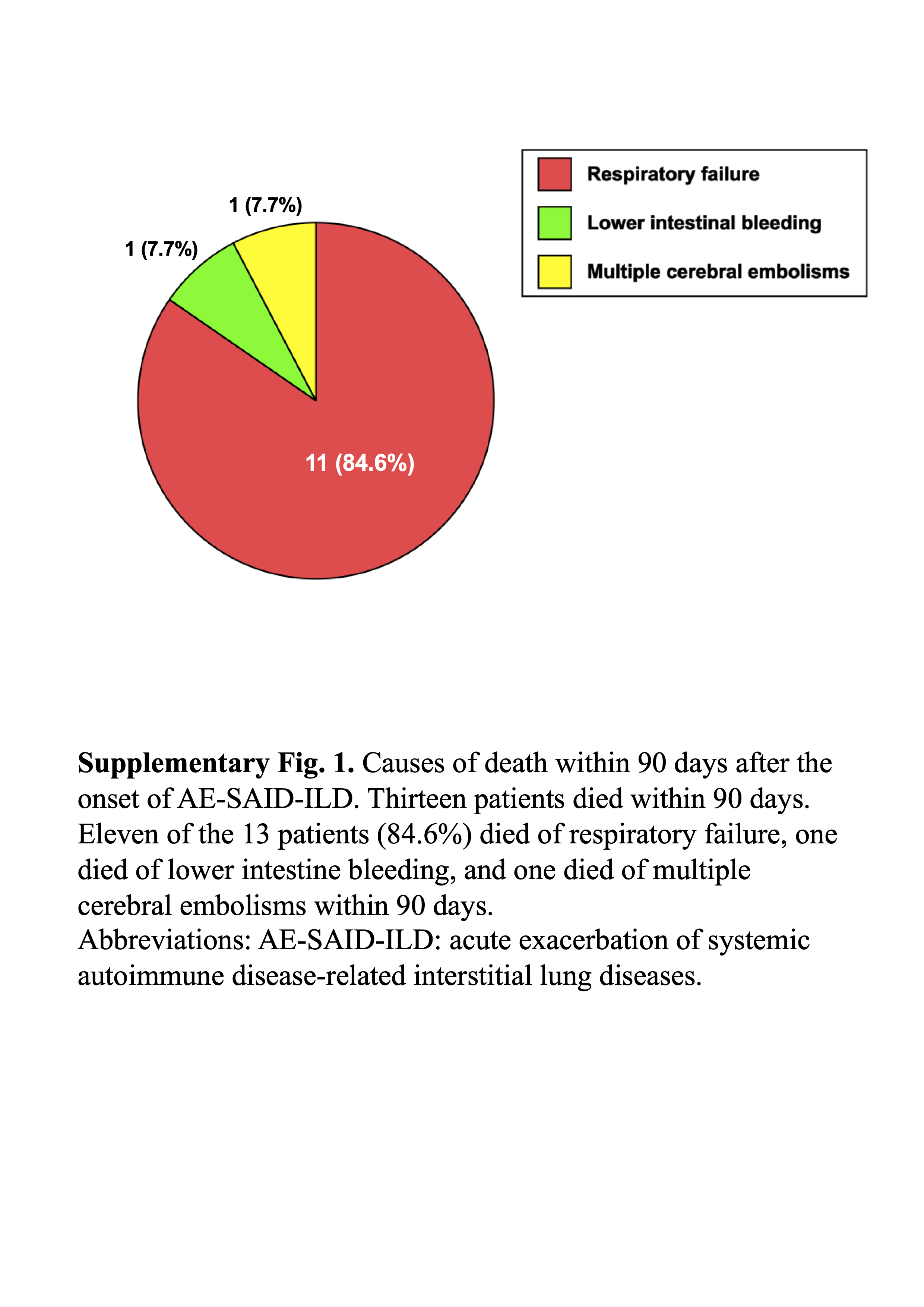

Supplement: Supplementary file 1 — Additional file1: Supplementary Fig. 1. Causes of death within 90 days after the onset of AE-SAID-ILD. Thirteen patients died within 90 days. Eleven of the 13 patients (84.6%) died of respiratory failure, one died of lower intestine bleeding, and the other died of multiple cerebral embolisms within 90 days. Abbreviations: AE-SAID-ILD: acute exacerbation of systemic autoimmune disease-related interstitial lung diseases. [file 12890_2022_2197_MOESM1_ESM.tiff]
